# Evaluating GPT-4V’s performance in the Japanese national dental examination: A challenge explored

**DOI:** 10.1016/j.jds.2023.12.007

**Published:** 2023-12-22

**Authors:** Masaki Morishita, Hikaru Fukuda, Kosuke Muraoka, Taiji Nakamura, Masanari Hayashi, Izumi Yoshioka, Kentaro Ono, Shuji Awano

**Affiliations:** a Division of Clinical Education Development and Research, Department of Oral Function, Kyushu Dental University, Kitakyushu, Japan; b Health Information Management Office, Kyushu Dental University Hospital, Kitakyushu, Japan; c Division of Maxillofacial Surgery, Department of Physical Function, Kyushu Dental University, Kitakyushu, Japan; d Division of Periodontology, Department of Oral Function, Kyushu Dental University, Kitakyushu, Japan; e Administration Department, Kyushu Dental University Hospital, Kitakyushu, Japan; f Division of Oral Medicine, Department of Physical Function, Kitakyushu, Japan; g Division of Physiology, Department of Health Promotion, Kyushu Dental University, Kitakyushu, Japan

**Keywords:** ChatGPT-4V, Image recognition, National dental examination, Medical image analysis

## Abstract

**Background/purpose:**

Rapid advancements in AI technology have led to significant interest in its application across various fields, including medicine and dentistry. This study aimed to assess the capabilities of ChatGPT-4V with image recognition in answering image-based questions from the Japanese National Dental Examination (JNDE) to explore its potential as an educational support tool for dental students.

**Materials and methods:**

The dataset used questions from the JNDE, which was conducted in January 2023, with a focus on image-related queries. ChatGPT-4V was utilized, and standardized prompts, question texts, and images were input. Data and statistical analyses were conducted using Qlik Sense® and GraphPad Prism.

**Results:**

The overall correct response rate of ChatGPT-4V for image-based JNDE questions was 35.0 %. The correct response rates were 57.1 % for compulsory questions, 43.6 % for general questions, and 28.6 % for clinical practical questions. In specialties like Dental Anesthesiology and Endodontics, ChatGPT-4V achieved correct response rates above 70 %, while response rates for Orthodontics and Oral Surgery were lower. A higher number of images in questions was correlated with lower accuracy, suggesting an impact of the number of images on correct and incorrect responses.

**Conclusion:**

While innovative, ChatGPT-4V’s image recognition feature exhibited limitations, especially in handling image-intensive and complex clinical practical questions, and is not yet fully suitable as an educational support tool for dental students at its current stage. Further technological refinement and re-evaluation with a broader dataset are recommended.

## Introduction

The development of large language models (LLMs) has recently accelerated, and the investigation of ways to utilize ChatGPT, developed by OpenAI, a well-known leader in LLMs, continues.^1^ Several studies have used ChatGPT to solve the text-based questions of national examinations for doctors, nurses, and pharmacists.^2^, ^3^, ^4^, ^5^ Because these studies were conducted when ChatGPT did not have an image recognition function, they only evaluated the usefulness of ChatGPT using textual information.^2^, ^3^, ^4^, ^5^

A new GPT-4 model with image recognition capability, GPT-4 with vision (GPT-4V), was released in September 2023.^6^ GPT-4V represents a major advancement of GPT-4, which was only able to understand and interpret information in a text-based manner. However, OpenAI has clarified that the image recognition capabilities currently available to the public are unsuitable for medical images.^6^ Nevertheless, it may be useful to evaluate the extent of GPT-4V’s current image recognition capabilities. The ability of GPT-4V to understand and interpret not only text but also image information to make decisions has important implications. In medicine, such systems are likely to be used in diagnosis and treatment of patients in the future. The Japanese National Dental Examination (JNDE) includes computed tomography (CT), magnetic resonance imaging (MRI), intraoral and extraoral photographs, diagrams, and tables, and evaluates examinees’ ability to integrate and judge the various types of information necessary for a dentist. The JNDE pass rate has decreased over time, and the examination is becoming more difficult every year.^7^ LLMs such as ChatGPT may be highly useful as educational support tools for dental students.

Although some studies have reported initial evaluations of GPT-4V’s image recognition capabilities,^8^, ^9^, ^10^, ^11^ there have been no reports of GPT-4V’s image recognition capabilities using national examination questions for any medical or dental profession. The present study was conducted to clarify the correct answer rate of GPT-4V, when its image recognition function was used to answer questions in the JNDE conducted in January 2023 involving information from various types of images, including figures and tables. We sought to examine the potential of GPT-4V as an educational support tool.

## Materials and methods

### Obtaining and processing data from the Japanese national dental examination

We downloaded a dataset containing questions and correct answers from the 116th National Dental Examination, administered in January 2023 in Japan, from the website of the Ministry of Health, Labour and Welfare (MHLW) of Japan.^12^ After the image recognition capability of ChatGPT-4 became standard for use after September 25, 2023,^6^ we extracted questions from the JNDE that included images such as intraoral and extraoral photographs, CT, MRI, echo, panoramic X-ray images, dental X-ray images, and photographs of dental technical work as well as diagrams, illustrations or tables, including polygonal analysis diagrams used in orthodontics. The MHLW scrutinizes the questions after administering the exam and discloses any inappropriate questions excluded from the exam results.^13^ A total of 160 questions were used in this study, excluding four inappropriate questions, seven compulsory questions, 55 general questions, and 98 clinical practical questions.

The Guidelines for the JNDE define the required basic topics, general dentistry, and each topic of dentistry.^7^ The required basic items are called “compulsory questions” and are considered to constitute the basic knowledge and skills necessary to become a dentist.^7^ The general dentistry and each topic of dentistry sections are based on the required basic knowledge and skills and are referred to as the General and Clinical Practical sections.^7^ The JNDE consists of three areas: compulsory questions, general questions, and clinical practical questions.^7^ The questions were subdivided by specialty using the National Dental Examination Question Book.^14^

### ChatGPT-4V

We used the ChatGPT-4V, September 25, 2023 model, in which image input is generally available to users.^1^,^6^ Standardized prompts and question text and images were entered into the web interface of ChatGPT, and all responses were recorded. Input was conducted from October 23 to 30, 2023. A prompt is intended to be “an instruction given to an LLM to enforce a rule, automate a process, or guarantee a specific quality and quantity of the generated output.”^15^ We standardized the format of the prompts when entering the question text and images as follows: “You are a student taking the Japanese National Dental Examination. Please answer the questions according to the question text and images. First, please indicate the correct answer choices. Then, please indicate your rationale for choosing the correct answer and your rationale for the errors in the other choices.”

### Data and statistical analysis

We utilized Qlik Sense® Enterprise August 2022 Patch 2 (Qlik Technologies, Inc., King of Prussia, PA, USA) for data analysis. We used GraphPad Prism 9.5.1 (GraphPad Software, Boston, MA, USA) for statistical analysis employing Mann-Whitney Tests.

## Results

**Figure 1:**
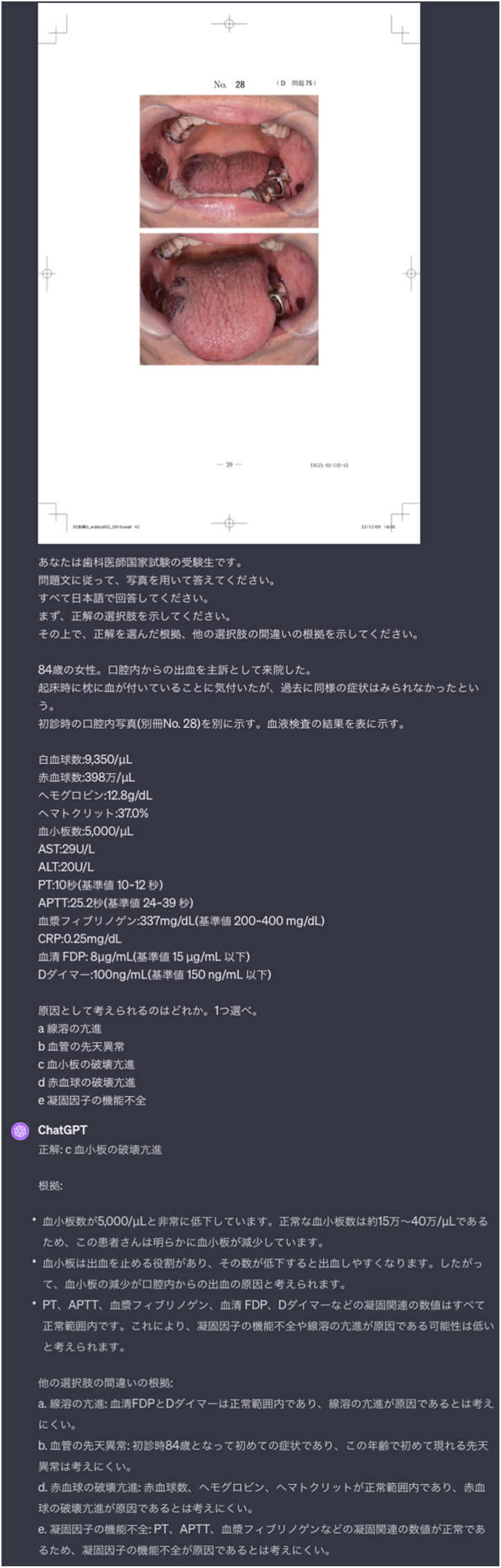
A screenshot depicting the process where question text and intraoral photographs from the Japanese national dental examination were entered into GPT-4V, resulting in the generation of responses.

Fig. 1 shows the interface of GPT-4V as it processes inputs from JNDE and illustrates a pivotal moment in the present study. The inputs include both textual questions and photographs. Upon submission of these inputs, the system generated corresponding responses.

Table 1A shows the percentage of correct answers for each of the compulsory, general, and clinical practical questions. The overall correct answer rate for the JNDE questions including images was 35.0 %. The compulsory question had the highest percentage of correct answers (57.1 %), followed by the general question (43.6 %) and the clinical practical question (28.6 %).

**Table 1:** The number of questions and percentage of correct answers by area, specialty, and number of specified correct answers in the Japanese National Dental Examination.

(A) Number of questions and percentage of correct answers by three areas of the Japanese National Dental Examination		
Area	Number of questions (n)	Correct answers (%)
Compulsory	7	57.1
General	55	43.6
Clinical practical	98	28.6
Total	160	35.0

(B) Number of questions and percentage of correct answers by each specialty.		
Specialty	Number of questions (n)	Correct answers (%)
Dental anesthesiology	4	75.0
Endodontics	7	71.4
Oral public health	8	50.0
Dental radiology	6	50.0
Dental materials	2	50.0
Pediatric dentistry	16	43.8
Oral surgery	34	38.2
Full denture	12	33.3
Restorative dentistry	13	30.8
Patrial denture	11	27.3
Orthodontics	20	25.0
Periodontics	13	23.1
Crown and bride	11	9.1
Dental anatomy	1	0.0
Oral physiology	1	0.0
Oral pathology	1	0.0

(C) Number of questions and percentage of correct answers by number of correct answers specified in the question.		
Number of specified correct answers	Number of questions (n)	Correct answers (%)
1	101	38.6
2	35	31.4
3	19	26.3
4	4	25.0
all	1	0.0

Table 1B shows the number of questions and the percentage of correct answers for each dental specialty, sorted from highest to lowest in terms of correct answers. The proportion of correct answers was over 70 % for Dental Anesthesiology and Endodontics. The correct response rate was 50 % for Oral Public Health, Dental Radiology, and Dental Materials. For Pediatric Dentistry, the rate of correct answers was in the 40–50 % range; for Oral Surgery, Complete Denture Dentistry, and Restorative Dentistry, the correct answer rate was in the 30–40 % range; for Partial Denture Dentistry, Orthodontics, and Periodontics, the correct answer rate was in the 20–30 % range. The correct answer rate was 9.1 % for Crown and Bridge Dentistry. There was only one question for each of Dental Anatomy, Oral Physiology, and Oral Pathology, comprising a total of three questions, all of which were answered incorrectly.

Table 1C shows the number of questions and the percentage of correct answers by the number of correct answers indicated in the question text. The number of questions by the number of correct answers indicated that the number of correct answers was highest for one question, and lowest for all. Similarly, the percentage of correct responses was highest for one question, at 38.6 %, followed by 31.4 %, 26.3 %, and 25.0 %, for two, three, and four questions, respectively, with the lowest percentage (0 %) for all.

After inputting the text and images of the questions, GPT-4V was unable to answer some questions. Of the 160 questions used in this study, 22 were unanswerable. Details were reviewed for the 22 questions that could not be answered. Of the 22 unanswerable questions, 4.5 % were general questions, and 95.5 % were clinical practical questions (Table 2A). Table 2B shows the dental specialties in order from highest to lowest percentage of correct answers. Orthodontics had the highest percentage at 36.4 %, followed by Oral Surgery at 27.3 %, Crown and Bridge Dentistry at 13.6 %, Pediatric Dentistry at 9.1 %, and Periodontics and Full and Partial Denture Dentistry at 4.5 % each. Table 2C shows the percentage of correct answers specified in the question text. The number of correct answers indicated in the question text was 2 for all of the unanswerable questions.

**Table 2:** The proportion of the 22 unanswerable questions by area, specialty, and number of specified correct answers.

(A) Proportion of questions by area in the 22 unanswerable questions.	
Area	Proportion (%)
General	4.5
Clinical practical	95.5
Total	100.0

(B) Proportion by specialty in the 22 unanswerable questions	
Specialty	Proportion (%)
Orthodontics	36.4
Oral surgery	27.3
Crown and bridge	13.6
Pediatric dentistry	9.1
Periodontics	4.5
Full denture	4.5
Partial denture	4.5
Total	100.0

(C) Proportion of correct answers by number of correct answers specified in the 22 unanswerable questions.	
Number of specified correct answers	Proportion (%)
2	100.0
Total	100.0

**Figure 2:**
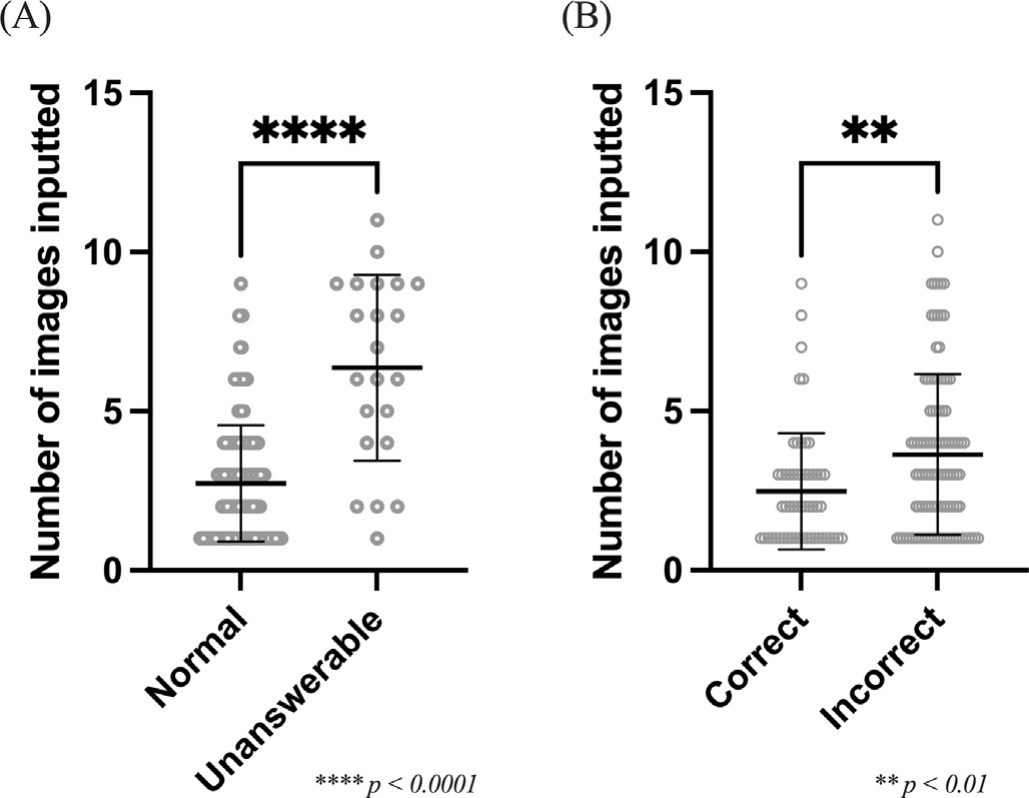
(A) Comparison of the number of input images between normal and unanswerable in 160 questions. (B) Comparison of the number of input images between correct and incorrect answers in 160 questions.

Fig. 2A shows a statistical evaluation of the number of images entered for the 138 questions that were successfully answered and the 22 questions that could not be answered. The results indicated that the number of images entered was significantly higher in cases of non-responses compared with cases of normal responses.

Fig. 2B shows a comparison of correct and incorrect answers for the number of images entered for the 160 questions in the present study. The results showed that the number of images entered was significantly higher for incorrect answers compared with correct answers.

## Discussion

Evaluation of the image recognition feature for GPT-4V, which was added as a new feature to ChatGPT-4 on September 25, 2023,^6^ for JNDE questions containing images, revealed a 35.0 % correct response rate for the 160 questions tested in this study. The compulsory questions are intended to represent the fundamental knowledge required of dentists, and the standard for passing these questions is 80 %. The 57.1 % correct response rate for the compulsory questions was higher than the low response rates for general questions and clinical practical questions (Table 1A).

According to OpenAI,^6^ the interpretation of medical images by GPT-4V is inconsistent, and the model sometimes gives accurate answers and sometimes gives incorrect answers to the same questions. Given the risks associated with the incomplete performance and inaccuracy of the model in this domain, the current version of GPT-4V is not considered to be suitable for performing medical functions. Based on our results, we do not believe that the current version of GPT-4V is suitable for performing medical functions or substituting professional dental advice, diagnosis, treatment, or judgment.

In JNDE, the general question and clinical practical question was more complicated than the compulsory question, and unsurprisingly, the percentage of correct answers to general question was lower than the percentage of correct answers to the compulsory question. Additionally, it is not surprising that the correct response rate for the clinical practical questions, which require reading and answering multiple items of imaging information, was also low, at 28.6 %. Regarding the percentage of correct responses by specialty, the image recognition function may perform better for Dental Anesthesiology and Endodontics. The percentage of correct responses may have been lower for oral surgery (38.2 %) because of the large number of images provided, including radiographic images, intraoral and extraoral photographs, CT, MRI, echo, and pathology, and the need to integrate a large amount of information to answer the questions. Similarly, Orthodontics questions also involved many images and a polygon table, which may have resulted in a low percentage of correct responses (25.0 %) because of the greater complexity of the information. Oral Anatomy, Oral Physiology, and Oral Pathology, all of which had a 0 % correct response rate, each included only one question, which may have impacted the correct response rate.

Regarding the number of correct answers indicated by the text of the question, a smaller number appeared to increase the percentage of correct answers. However, the reason for the decrease in the percentage of correct remains to be clarified, although the number of correct answers of 4 is the same as choosing one that is incorrect. It should also be noted that the instruction to “choose all” was difficult even for human examinees,^16^ and GPT-4V exhibited similar difficulty answering correctly. The inclusion of only one question may have had an effect.

Most of the 22 questions GPT-4V was unable to answer were clinical practical questions, possibly because Orthodontics and Oral Surgery are areas that require answers based on the integration of a large number of images. Prosthodontics, such as Crown and Bridge, Full Denture, and Partial Denture, accounted for 22.6 % of the total, suggesting that GPT-4V’s image recognition function may not be effective for answering prosthodontics-related questions. In addition, Periodontics included a Periodontal Tissue Examination Table, which may be a weak point for the image recognition function.

The number of correct answers in the question text was 2 in all 22 unanswerable questions. Determining the reason for this finding will require further investigation. The number of images entered at the same time as the question text may affect the rates of correct and incorrect answers, and statistical evaluation revealed that the number of images for incorrect answers was significantly greater than that for correct answers, suggesting that the GPT-4V image recognition function was more likely to obtain correct answers when fewer images were entered. A comparison of the number of images entered for correct and incorrect answers, respectively, shows that significantly more images were entered for incorrect answers, suggesting that reducing the number of images entered may be necessary to obtain correct answers with the image recognition function.

A limitation of the current study is that the analysis was conducted on questions from a single national dental examination, and the results may be biased because of the small number of questions in each specialty. Care should be taken in interpreting the results obtained in this study, because ChatGPT is constantly being updated with new features and machine learning content, and the results obtained in the current study may differ from those obtained with the same data set 1 year later. As OpenAI points out, GPT-4V may produce different results from multiple inputs, even for the same question or image.^6^ Another new feature of GPT-4V is that its image recognition function is unsuitable for discriminating medical images.^6^ An additional limitation of the current study is that no previous studies have evaluated national examination questions containing images with GPT-4V’s image recognition function using a similar approach to that used in this study. Therefore, we cannot present an in-depth discussion of the current study in relation to similar research.

In conclusion, the current evaluation of ChatGPT-4V’s image recognition capabilities revealed significant limitations in the context of the JNDE. The overall correct response rate for image-based questions was 35.0 %. These findings indicate that, although ChatGPT-4V’s image recognition feature is innovative, it is not yet sufficiently reliable or comprehensive for use as an educational tool in the medical and dental fields. Further technological advancements and comprehensive evaluations with broader datasets will be required to enhance the system’s applicability in clinical and educational settings.

## Declaration of competing interest

The authors have no conflicts of interest relevant to this article.
